# Older age and post-traumatic organ failure a TraumaRegister DGU^®^ analysis of 34,469 elderly major trauma patients

**DOI:** 10.1007/s00068-026-03240-2

**Published:** 2026-06-15

**Authors:** Tim Niklas Bewersdorf, Rolf Lefering, Stephan Stein, Jan Streblow, Sebastian Findeisen, Gerhard Schmidmaier, Tobias Grossner

**Affiliations:** 1 Faculty of Medicine, Heidelberg University, 69120 Heidelberg, Germany; 2 Heidelberg Trauma Research Group, Clinic for Trauma and Reconstructive Surgery, Centre for Orthopaedics, Trauma Surgery and Spinal Cord Injury, University Hospital Heidelberg, 69120 Heidelberg, Germany; 3 Institute for Research in Operative Medicine (IFOM), University of Witten / Herdecke, Cologne, Germany

**Keywords:** Post-traumatic organ failure, Post-traumatic multiple organ failure, Elderly major trauma patients, Mortality risk factors, TraumaRegister DGU^®^

## Abstract

**Purpose:**

As the number of elderly major trauma patients (EMTP) increases continuously, this study sought to determine the incidence of organ failure (OF) and multiple organ failure (MOF) and their impact on mortality and recovery status in this cohort.

**Methods:**

Based on the German TraumaRegister DGU^®^ database this study included EMTP aged between 65 and 99 years with an Injury Severity Score (ISS) of ≥ 16 between 2014 and 2023. OF was defined as Sequential Organ Failure Assessment (SOFA) score of ≥ 3 and MOF as failure of ≥ 2 organs.

**Results:**

The database revealed 34,469 EMTP, here 37% suffered from OF (16.0%) or MOF (21.0%). Mortality was significantly higher in OF and MOF patients compared to non-OF patients (non-OF: 7.4%; OF: 45.0%; MOF: 59.0%; *p* < 0.001). 80.9% of all deaths were associated with failure of at least one organ and 51.1% of non-survivors suffered from MOF. Highest odds ratios (OR) for mortality were found for liver (OR: 5.13, *p* < 0.001), central nervous system (OR: 3.63, *p* < 0.001) and renal failure (OR: 3.25, *p* < 0.001), followed by cardiovascular (OR: 1.95, *p* < 0.001) and lung failure (OR: 1.16, *p* = 0.010). Haemostatic failure had no impact on mortality.

**Conclusion:**

OR and mortality differ significantly between distinct OFs due to varying degrees of success in treatment options and systemic impact of these OFs. Clinicians should be aware that OF and MOF occur frequently in EMTP and that these patients have a high risk of dying. Therefore, close screening for and prompt treatment of OF and MOF could be a key strategy to lower mortality of EMTP.

**Supplementary Information:**

The online version contains supplementary material available at10.1007/s00068-026-03240-2.

## Introduction

Due to increasing life expectancy the number of elderly major trauma patients (EMTP) has risen disproportionately in recent decades [1–5]. The improvements in treatment of chronic conditions, like cardiovascular diseases, diabetes, or kidney diseases, allows patients to live longer actively and in good health [5, 6]. Consequently preexisting comorbidities and polypharmacy among elderly trauma patients are increasing [5]. The high prevalence of comorbidities, chronic impaired organ function and increased frailty result in a limited physiological reserve of several critical organs and a consecutive higher risk of organ failure (OF) and multiple organ failure (MOF) after sustaining major trauma [7–9].

Over several decades preclinical and clinical resuscitation of major trauma patients has been improving continuously allowing more patients to survive the initial phase after trauma [3, 8, 9]. Among survivors of the initial phase MOF is the leading cause of late death, with a peak around 14 days after trauma, with a reported mortality up to 50% [9–11]. Therefore, OF and MOF remains a major challenge highlighting the importance of a specialized risk stratification and complication management especially when treating EMTP, who are at higher risk of developing an OF or MOF [1, 2, 4, 7]. As OF and MOF in EMTP increase hospital and intensive care unit (ICU) length of stay (LoS) significantly and worsen functional outcomes and recovery of survivors, this also poses a significant financial burden [8, 10, 12, 13].

Precise understanding of risk of death in trauma patients is essential for an appropriately management of these patients. Therefore several scores, like the Injury Severity Score (ISS), the Trauma and Injury Severity Score (TRISS) or the Revised Injury Severity Classification (RISC) score, have been implemented during the last decades [14–16]. These scores aim to provide a better comparability between the heterogenous case mix and injury pattern of major trauma patients. Despite revisions of these scores, like the New Injury Severity Score (NISS) or the RISC II score, these scores do not take late complications like OF and MOF into account for prediction of mortality, as their intention is to predict the mortality shortly after admission [17, 18].

However, there remain significant knowledge gaps regarding prevalence, risk factors and outcomes of distinct OF and MOF types in EMTP, as only a few studies investigated OF and MOF in this patient cohort. Therefore, this study evaluates prevalences of distinct OFs and MOF among different age groups in EMTP and identified the impact of OF and MOF on the outcome parameters mortality, LoS at ICU and recovery status upon discharge.

## Methods

### TraumaRegister DGU^®^

This retrospective, observational, nationwide, register-based cohort study is based on data of the TraumaRegister DGU^®^ (TR-DGU; https://www.auconline.de/unsere-angebote/medizinische-register/traumaregister-dgu/) database of the German Trauma Society (Deutsche Gesellschaft für Unfallchirurgie, DGU). The general objective of this multi-centre registry is to gather pseudonymised and standardised pre-clinical and clinical data of severely injured trauma patients. For each patient, detailed information is recorded, such as demographic characteristics, injury patterns, pre-injury American Society of Anesthesiologists (ASA) score, as well as preclinical treatment and treatment in the trauma resuscitation room (TRR), operating theatre and at ICU. The documentation also covers important laboratory results, blood transfusions, complications and the outcome of each individual. All trauma patients admitted to the hospital via TRR with subsequent ICU care or trauma patients reaching the hospital with vital signs but die before admission to ICU are documented within the TR-DGU.

In general, the quality of the registry data is high, and rate of missing data is low [19]. All scientific analyses of the data must be approved through a peer review process, as specified in the publication guidelines of the TR-DGU. Further information about the TR-DGU is provided in the supplemental file.

### Patients

**Fig. 1:**
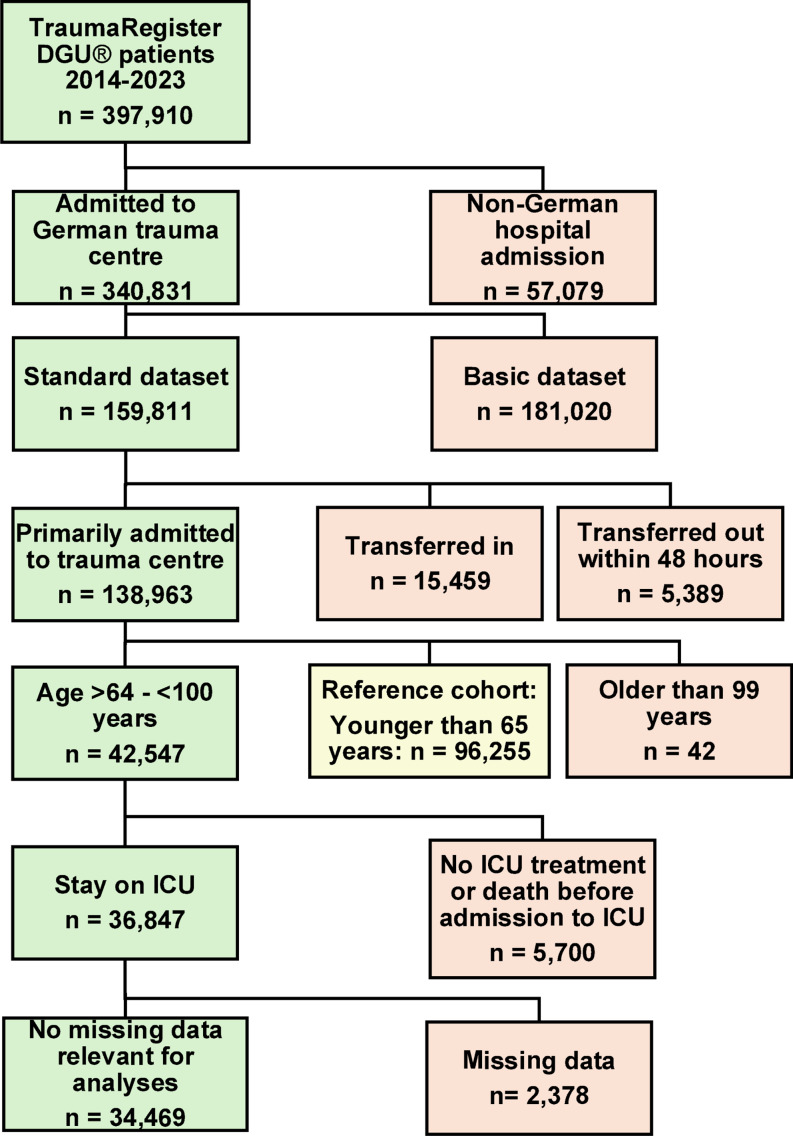
Inclusion flow of selected patients from the TraumaRegister DGU^®^ (TR-DGU)

For this study (project-ID: TR-DGU 2024-044), data from trauma patients admitted to a German trauma centre within a 10 years’ period from 2014 to 2023 were included in the analyses. Further selections were performed as displayed in the flow chart (Fig. 1) to provide high-quality and homogenous data. As OF is solely documented within the standard form of the TR-DGU, only patients assessed with standard datasets were included. Patients, who were transferred out within the first 48 h, were admitted from another hospital or were not treated in the TRR, were excluded. From this population we excluded all patients under the age of 65 or older than 99 years, as this cohort was comparatively small (*n* = 42). Furthermore, patients without stay at ICU were also excluded from the study, because these patients were either slightly injured or died before admission to ICU and therefore died before OF and MOF occur. This left 36,847 patients out of 340,831 documented major trauma patients, treated in 242 trauma centres in Germany within 2014 to 2023. Out of these 36,847 patients another 2,378 patients were excluded due to missing data about OF. Consequently, 34,469 patients were selected for data analysis. To contextualise incidences of MOF and distinct OFs among EMTP, we compared incidence rates of EMTP with those of patients aged 18–64 years, in which the same exclusion criteria were applied. Therefore, these patients aged 18–64 years served as a reference group (Fig. 1).

### Organ failure and multiple organ failure

Newly diagnosed OF, MOF and sepsis, which occurred post-injury, were reported in the registry’s binary system (yes/no). OF was documented according to the Sequential Organ Failure Assessment (SOFA) score 3 or 4 of the following six organ systems: lung/respiration, coagulation, cardiovascular system, liver, kidney, and the central nervous system (CNS) [20]. SOFA score was assessed every day and the worst daily value for each organ system was documented [20]. As the original SOFA score published by Vincent et al. did not define MOF [20, 21], MOF was defined in this study as failure (SOFA score ≥ 3) of two or more organs either on the same day or different days during stay on ICU. Detailed information is displayed in Table 1.

**Table 1:** Detailed information about organ failure assessment using the Sequential Organ Failure Assessment (SOFA) score published by Vincent et al. [20]

Organ	Parameter	Unit	Score			
			1	2	3	4
Lung / respiration	PaO_2_/FiO_2_	mmHg	< 400	< 300	< 200 with ventilation	< 100 with ventilation
Coagulation	Thrombocytes	1,000/mm^3^	< 150	< 100	< 50	< 20
Cardio-vascular system	Blood pressure or catecholamines	mmHg	MAP < 70	Catecholamines low	Catecholamines intermediate	Catecholamines high
Liver	Bilirubin	mg/dl	1.2–1.9	2.0-3.4	6.0-11.9	≥ 12.0
Kidney	Creatinine or urine output	mg/dl ml/day	1.2–1.9	2.0-3.4	3.5–4.9 < 500	≥ 5.0 < 200
Central nervous system	Glasgow Coma Scale		14 − 13	12 − 10	9 − 6	< 6

SOFA score 1–2: organ dysfunction; SOFA score 3–4: organ failure

### Injury severity and further parameters

All injuries documented in the TR-DGU database were coded according to the Abbreviated Injury Scale (AIS), and injury severity was assessed by calculating the Injury Severity Score (ISS), which ranges from 1 to 75 points with higher scores indicating worse injury [14]. The injury severity of a body region was defined as the worst AIS severity level of all injuries in that body region and was reported as maximum AIS (MAIS).

Major trauma patients are a very heterogenous population and therefore mortality prediction is quite complicated. Pre-injury comorbidity status was evaluated by using the ASA score. To assess the impact of OF and MOF on the mortality, it is important to predict the baseline mortality if the patient would not suffer from OF or MOF. The RISC II score, which takes injury severity, age, sex, pre-injury ASA score, physiological reaction to trauma and laboratory values into account, can predict mortality after trauma shortly after admission. The score, which ranges from − 6 (99% mortality) to 0 (50% mortality) to + 6 (1% mortality), has been developed and validated using TR-DGU data [17]. As the RISC II score is superior in baseline mortality prediction to other scores, like RISC, TRISS, ISS and NISS it was used to predict baseline mortality in EMTP within this study [17]. Moreover, the RISC II score was used for adjustment of differing injury severities and patterns as it takes the two most severe injuries (AIS ≥ 3), as well as severe head injuries (AIS 3–4) and critical head injuries (AIS 5–6) into account. This adjustment reflects the higher mortality rates in AIS ≥ 3 head injuries in comparison to AIS ≥ 3 injuries of other body regions. As the RISC II score also considers the ASA score a further adjustment for the pre-injury comorbidity status was performed. Further parameters considered in a multivariate logistic regression were different sites of OFs, MOF, sepsis and blood transfusion before ICU admission, as these findings were reported to be additional risk factors for higher mortality and prolonged ICU stay [1, 12].

### Endpoints and statistics

The co-primary endpoints of this study were the incidence of distinct OF and MOF and its effects on 30-day inpatient mortality among EMTP. We performed a multivariate logistic regression to identify risk factors of 30-day inpatient mortality. Secondary outcomes included LoS at ICU and the recovery status at time of hospital discharge as assessed by the Glasgow Outcome Scale (GOS), which is a wide-spread recovery status assessment tool in major trauma patients and reaches from 1 point (death) / 2 points (coma) to 5 points (full recovery) [22, 23]. Therefore, we also evaluated effects of OF and MOF on LoS at ICU and recovery status upon discharge as secondary endpoints.

Descriptive analysis is reported as absolute numbers with percentages for categorical variables, or as means with standard deviation (SD) for numerical variables. LoS at ICU and LoS at the hospital are presented as medians with interquartile ranges (IQR). ISS is presented both with means (+ SD) and medians (+ IQR) to provide an optimal interpretability. For statistical analyses data were examined using Chi-square-test for binary data and Mann-Whitney-U-test for ordinal or metric data, as well as Spearman correlation. Given the large sample size, p-values should be interpreted cautiously as even marginal clinical differences can be highly significant. The effect of various types of organ failure was analysed using a multivariate logistic regression with hospital mortality as dependent variable. RISC II score, blood transfusion before ICU admission, sepsis and the presence of MOF served as independent variables. The RISC II score served as a descriptor for the cumulated risk of death on admission. Results are presented as odds ratios (OR) with 95%-confidence intervals. Statistical significance was set at *p* ≤ 0.05. SPSS statistics (version 29, IBM Inc., Armonk NY, United States) was used for all statistical analyses.

## Results

### General findings

As displayed in Fig. 1, during the 10-year-period of 2014 to 2023, 397,910 patients were documented in the TR-DGU. Among these patients 42,547 patients aged 65–99 years were primarily admitted to a German TRR and were documented using the TR-DGU standard form. After application of all exclusion criteria 34,469 patients were included in this study. As displayed in Table 2 the average age in the study population was 77.6 years and 60.4% were male.

**Table 2:** Patients’ demographics, injury severity, therapy and outcome distinguished by different organ failures (OF), multiple organ failures (MOF) and non-OF

Specific OF	MOF	OF	Non-OF	Total
*n*	7,239	5,528	21,702	34,469
Prevalence within the study cohort	21.0%	16.0%	63.0%	-
Age *	78.0 (7.4)	78.0 (7.7)	77.2 (7.7)	77.6 (7.7)
Males	4,731 (65.4%)	3,365 (60.9%)	12,725 (58.6%)	20,821 (60.4%)
ASA ≥ 3	3,612 (55.0%)	2,712 (49.1%)	8,288 (38.2%)	14,612 (42.4%)
Injury Severity Score (ISS) mean*	27.7 (13.3)	23.2 (11.3)	15.5 (9.2)	19.3 (11.7)
Injury Severity Score (ISS) median**	25 (18–34)	25 (16–29)	14 (9–21)	17 (10–25)
MAIS head ≥ 3	5,004 (69.1%)	3,679 (66.6%)	8,208 (37.8%)	16,891 (49.0%)
MAIS thorax ≥ 3	3,251 (44.9%)	1,815 (32.8%)	7,195 (33.2%)	12,261 (35.6%)
MAIS abdomen ≥ 3	675 (9.3%)	295 (5.3%)	866 (4.0%)	1,836 (5.3%)
MAIS extremities ≥ 3	1,667 (23.0%)	976 (17.7%)	3,302 (15.2%)	5,945 (17.2%)
Blood transfusion prior to ICU admission	1,474 (20.4%)	565 (10.2%)	676 (3.1%)	2,715 (7.9%)
Mortality^†^	4,269 (59.0%)	2,490 (45.0%)	1,597 (7.4%)	8,356 (24.2%)
Length of stay at ICU (days)**	9 (3–20)	5 (2–13)	2 (1–5)	3 (1–8)
Glasgow Outcome Scale (GOS) ***: good recovery (5)	511 (17.5%)	1,035 (34.7%)	12,906 (65.0%)	14,452 (56.1%)
GOS ***: moderate disability (4)	951 (32.6%)	1,069 (35.8%)	5,529 (27.8%)	7,549 (29.3%)
GOS ***: severe disability (3)	1,149 (39.4%)	725 (24.3%)	1,338 (6.7%)	3,212 (12.5%)
GOS ***: coma (2)	308 (10.6%)	157 (5.3%)	97 (0.5%)	562 (2.2%)

MAIS: Maximum Abbreviated Injury Scale; ICU: intensive care unit. *: mean and standard deviation. **: median and interquartile range. ***: recovery status of survivors according to the Glasgow Outcome Scale (GOS): good recovery: resumption of normal life; moderate disability: patient independent in daily life; severe disability: patient dependent for daily support; coma: persistent neurovegetative state, patient unresponsive [23]. ^†^: *p* < 0.001 across all groups (Chi-square-test)

Mean ISS was 19.3 (SD: 11.7) and 42.4% suffered from relevant comorbidities (ASA score 3 or 4), which resulted in mean inpatient mortality within 30 days of 24.2%. With increasing age, mortality constantly increased with lowest mortality in patients aged 65 to 69 (11.3%) and highest mortality in patients aged 90 or above (42.1–43.5%), although injury severity according to ISS remained stable in patients aged between 65 and 94 and was lower in patients aged 95–99 years (Table 3). The head was the anatomical region, in which a severe injury (MAIS ≥ 3) was observed most frequently (49.0%), followed by the thorax (35.6%), while the anatomical regions abdomen and extremities showed less frequently serious injuries (extremities: 17.2%; abdomen: 5.3%; Table 2).

**Table 3:** Percentage, injury severity and mortality of different age cohorts

Age	*n*	Mean ISS	Mortality
65–69	6,605	18,9	11.3%
70–74	6,122	19,4	16.1%
75–79	7,370	19,4	21.8%
80–84	7,362	19,7	29.9%
85–89	4,717	19,1	38.7%
90–94	1,911	19,0	43.5%
95–99	382	17,0	42.1%
All patients	34,469	19.3	24.2%

ISS: Injury Severity Score

### Organ failure and multiple organ failure

In general, MOF (21.0%) was detected more often than single OF (16.0%) in this study. Prevalences of OF and MOF among EMTP were higher than in the reference cohort of patients aged between 18 and 64 years (OF: 9.9%, MOF: 12.8%). CNS failure was the most frequently documented isolated OF, followed by cardiovascular failure (Table 4). As the cardiovascular system can affect several functions of other organs, cardiovascular failure was the most common OF in patients suffering from MOF (84.7%), followed by CNS failure (73.2%).

**Table 4:** Percentage of distinct organ failures (OF) on isolated OF and multiple organ failure (MOF)

	Percentage within isolated OFs	Percentage within MOF
*n*	5,528	7,239
CNS failure	43.3%	73.2%
Cardiovascular failure	32.2%	84.7%
Lung failure	10.0%	54.7%
Haemostatic failure	9.5%	26.5%
Renal failure	4.8%	22.1%
Liver failure	0.2%	5.7%

CNS: central nervous system

**Fig. 2:**
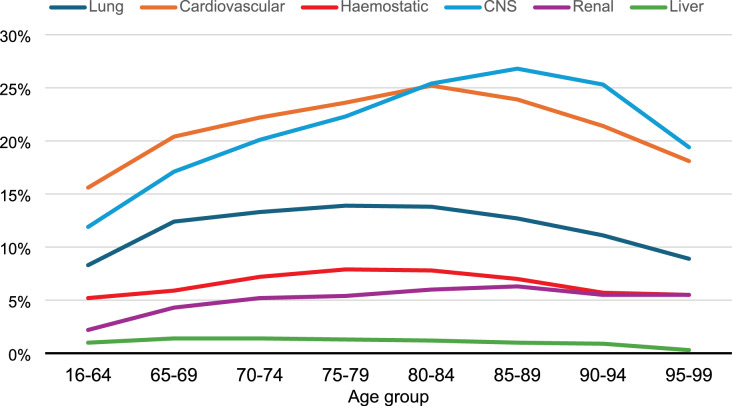
Prevalence of a specific organ failure divided by age after major trauma

Prevalence of a specific OF in different age groups showed bell-shaped patterns with decreasing prevalence in the oldest age groups, as displayed in Fig. 2. While the highest overall prevalence in the study population was observed for cardiovascular failure (23.0%), the prevalence of CNS failure among patients aged 85–89 years was the highest detected prevalence (26.8%). Earliest drop after prevalence peak was observed for liver failure, as the highest prevalence of liver failure was in the cohort 65–69 years old, followed by lung and haemostatic failure with highest prevalences in the 75–79 years old cohort. While prevalence peak of cardiovascular failure was detected in the 80–84 years old cohort, latest drop after prevalence peak was detected for CNS and renal failure with highest prevalence in the group aged 85–89 years and were therefore detected at least 10 years later than liver, lung and haemostatic failure.

In general, 51.1% of non-survivors suffered from MOF, while 29.8% suffered from isolated OF, resulting in only 19.1% of all deaths occurred without any association to OF and MOF. Distinct OFs showed significant differences in mortality (*p* < 0.001), as the lowest mortality was found in haemostasis (49.6%), followed by lung (51.6%), cardiovascular (52.5%) and renal failure (59.8%), while highest mortality rates were detected for CNS (65.2%) and liver failure (77.3%) (Table 5).

**Table 5:** Patients’ demographics, injury severity, therapy and outcome distinguished by different organ failures (OF), multiple organ failures (MOF) and non-OF

Specific OF	CNS failure	Cardiovascular failure	Lung failure	Haemostatic failure	Renal failure	Liver Failure
*n*	7,696	7,911	4,514	2,440	1,867	423
Prevalence within the study cohort	22.3%	23.0%	13.1%	7.1%	5.4%	1.2%
Number/percentage of isolated OF in specific OF	2,395 (31.1%)	1,781 (22.5%)	551 (12.2%)	523 (21.4%)	266 (14.2%)	12 (2.8%)
Age *	78.7 (7.5)	77.9 (7.5)	77.6 (7.4)	77.8 (7.3)	78.5 (7.5)	76.7 (7.2)
Males	4,807 (62.5%)	5,118 (64.7%)	3,085 (68.3%)	1,501 (61.5%)	1,270 (68.0%)	278 (65.7%)
ASA 3/4	3,780 (49.1%)	3,890 (49.2%)	2,200 (48.7%)	1,255 (51.4%)	1,084 (58.1%)	215 (50.8%)
Injury Severity Score (ISS) mean*	27.4 (12.7)	26.7 (13.4)	27.4 (13.4)	27.9 (13.9)	25.8 (14.2)	28.6 (14.4)
ISS median**	25 (20–33)	25 (17–34)	25 (18–34)	25 (17–35)	25 (16–34)	27 (17–38)
MAIS head ≥ 3	6,454 (83.9%)	5,051 (63.8%)	2,899 (64.2%)	1,329 (54.5%)	887 (47.5%)	191 (45.2%)
MAIS thorax ≥ 3	2,592 (33.7%)	3,593 (45.4%)	2,285 (50.6%)	1,203 (49.3%)	985 (52.8%)	240 (56.7%)
MAIS abdomen ≥ 3	402 (5.2%)	762 (9.6%)	312 (6.9%)	368 (15.1%)	291 (15.6%)	95 (22.5%)
MAIS extremities ≥ 3	1,111 (14.4%)	1,996 (25.2%)	1,096 (24.3%)	876 (35.9%)	602 (32.2%)	176 (41.6%)
Blood transfusion prior to ICU admission	1,117 (14.5%)	1,589 (20.1%)	881 (19.5%)	774 (31.7%)	479 (25.7%)	171 (40.4%)
Mortality with isolated OF	1,707 (71.3%)	498 (28.0%)	159 (28.9%)	62 (11.9%)	60 (22.6%)	4 (33%)
Mortality within OF and MOF^*†*^	5,018 (65.2%)	4,155 (52.5%)	2,329 (51.6%)	1,211 (49.6%)	1,117 (59.8%)	327 (77.3%)
Length of stay at ICU (days)**	5 (2–15)	8 (3–18)	12 (4–23)	8 (2–19)	10 (4–23)	11 (3–23)

CNS: central nervous system; MAIS: Maximum Abbreviated Injury Scale; *ICU: intensive care unit.* *: mean and standard deviation. **: median and interquartile range. ^†^: *p* < 0.001 across all organ failure types (Chi-square-test)

**Fig. 3:**
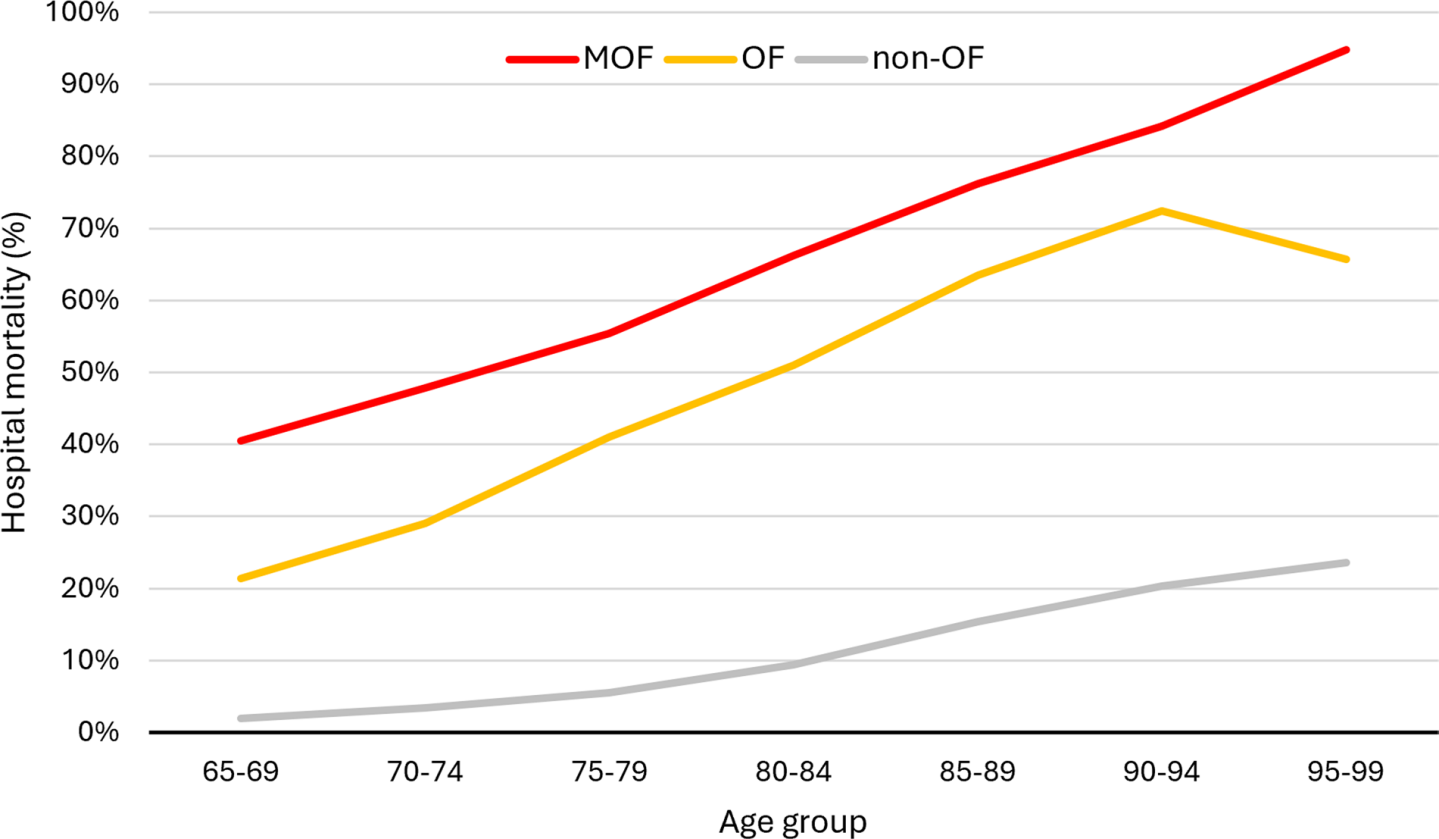
Mortality of elderly major trauma patients suffering from multiple organ failure (MOF), organ failure (OF) or no organ failure (non-OF) in age subgroups

Detailed analysis of mortality rates divided by age subgroups showed increasing mortality with increasing age for non-OF, OF and MOF patients, except OF in the oldest subgroup compared to the second oldest subgroup (Fig. 3). Highest mortality rate was detected for MOF among patients aged 95–99 years with an average mortality of 94.8%, while highest mortality in OF patients was 72.4% and 23.6% in patients without any OF. Increase of mortality rates with increasing age showed a continuously steeper increase in OF and MOF patients compared to non-OF patients, as displayed in Fig. 3.

OF and MOF were associated with higher injury severity (Table 6) shown by a correlation of ISS with number of OFs (Spearman correlation: *r* = 0.44, *p* < 0.001). With rising numbers of OFs, mortality increased significantly resulting in a mean mortality of MOF patients of 59.0%. Patients with OF and MOF were more often male (males: non-OF: 58.6%, OF: 60.9%, MOF: 65.4%), but there was no difference in mean age between non-OF, OF and MOF among elderly patients.

**Table 6:** Percentage, injury severity and mortality of number of organ failures

Number of OFs	*n*	ISS (standard deviation, *p*-value compared to previous group*)	Mortality
0	21,702 (63.0%)	15.5 (9.2)	7.4%
1	5,528 (16.0%)	23.2 (11.3, *p* < 0.001)	45.0%
2	3,944 (11.4%)	26.2 (12.5, *p* < 0.001)	56.3%
3	2,118 (6.1%)	28.4 (13.7, *p* < 0.001)	58.7%
4	847 (2.5%)	30.5 (14.2, *p* < 0.001)	63.0%
5	242 (0.7%)	32.6 (14.8, *p* = 0.040)	74.8%
6	88 (0.02%)	32.5 (16.5, *p* = 0.815)	88.6%
MOF	7,239 (21.0%)	27.7 (13.3)	59.0%
All patients	34,469	19.3 (11.7)	24.2%

ISS: Injury Severity Score; OF: organ failure; MOF: multiple organ failure. *: p-values were calculated using the Mann-Whitney-U-test

As mortality rates differed significantly between different OFs and mortality increased with the number of OFs, we compared different combinations of failed organs within MOF patients with a failure of solely two organs. Among these 3,944 patients with a combination of exactly two OFs the mean mortality was 56.3%. The highest mortality was detected in the combination of CNS and liver OF (87.5%), followed by CNS and haemostatic OF (68.5%). The combination of lung and haemostatic OF was associated with the lowest mortality rate (24.3%), which was comparable to the mortality of the whole cohort (24.2%), but lower than average mortality of OF (45.3%).

**Fig. 4:**
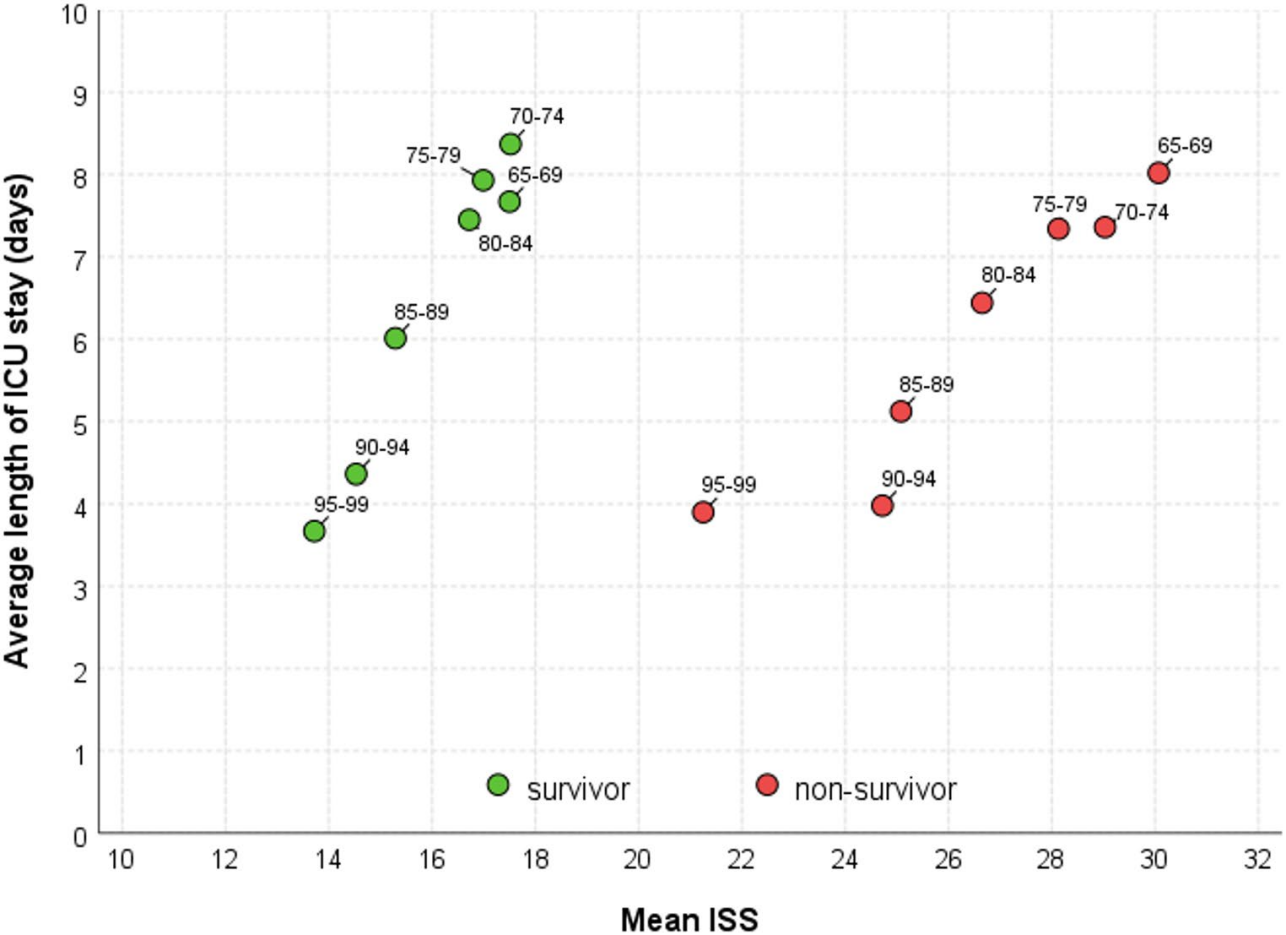
Mean length of stay at ICU and injury severity according to Injury Severity Score (ISS) of elderly major trauma patients divided by 5-year age cohorts. The number indicates the youngest age in the cohort. Green: survivors, red: non-survivors

Duration of ICU stay was substantially longer in OF and MOF patients (mean ICU LoS: non-OF: 2 days, OF: 5 days, MOF: 9 days, Table 2). In detail, we detected distinct differences in ICU LoS between different OFs with longest LoS at ICU documented in lung failure (12 days), liver failure (11 days) and renal failure (10 days) (Table 5). With increasing age ICU LoS and ISS decreased constantly both in survivors and non-survivors (Fig. 4). Except patients aged 65–69 and 95–99 years, non-survivors had shorter ICU LoS than survivors.

Status of recovery upon discharge from the hospital revealed drastically worse outcomes for MOF and OF survivors compared to patients without OF (Table 2). While 92.8% of non-OF were discharged with a favourable status of recovery of GOS ≥ 4, only 70.5% of all OF patients and 50.1% of all MOF patients had a GOS ≥ 4 upon hospital discharge. Consequently, 49.9% of MOF patients and 29.6% of OF patients needed daily or 24/7 support after hospital discharge.

### Impact of (multiple) organ failure on mortality

The additional effect on mortality of different types of OF was assessed by a multivariate logistic regression analysis where the RISC II score served as a descriptor of the basic mortality risk on admission. The results showed an increasing risk of death by all OFs, except haemostatic failure (Table 7). Highest ORs were found for liver failure (OR: 5.13, *p* < 0.001), followed by CNS failure (OR: 3.63, *p* < 0.001) and renal failure (OR: 3.25, *p* < 0.001). MOF and sepsis did not add an additional risk to the summarized risk of any specific OFs, as MOF and sepsis showed ORs < 1 (MOF: OR: 0.82, *p* = 0.013; sepsis: OR: 0.85, *p* = 0.002). Failure of the haemostatic system showed no significant impact on mortality (OR: 1.07, *p* = 0.299), while blood transfusion prior to ICU admission lowered mortality as OR was 0.64 (*p* < 0.001).

**Table 7:** Multivariate logistic regression for mortality risk modification after major trauma

	Odds ratio	Significance [*p*]	95%-confidence interval
RISC II prognostic score (per one point)	0.48	< 0.001	0.47–0.49
Haemostatic failure	1.07	0.299	0.94–1.22
Lung failure	**1.16**	**0.010**	1.04–1.30
Cardiovascular failure	**1.95**	**< 0.001**	1.75–2.17
Renal failure	**3.25**	**< 0.001**	2.81–3.74
CNS failure	**3.63**	**< 0.001**	3.31–3.99
Liver failure	**5.13**	**< 0.001**	3.78–6.97
Multiple organ failure	0.82	0.013	0.70–0.96
Sepsis	0.85	0.002	0.75–0.97
Blood transfusion before ICU admission	0.64	< 0.001	0.57–0.72

CNS: central nervous system; ICU: intensive care unit

## Discussion

### Organ failure and multiple organ failure

This study analysed the incidences of distinct OFs and MOF and their impact on mortality and morbidity in EMTP to gain a better understanding of OF and MOF in EMTP, since OF and MOF are leading causes of late death in major trauma patients [9–11].

We showed that 37.0% of all EMTP suffered from OF or MOF (OF: 16.0%; MOF: 21.0%), which is higher than the rates of OF and MOF among younger patients (18–64 years: OF: 9.9%, MOF: 12.8%), emphasizing that age, frailty and chronic organ dysfunctions are well-known patient-related risk factors for developing OF and MOF [5, 9, 24–26]. After increasing prevalence for all OFs with increasing age in younger subgroups and a prevalence peak in different age groups, prevalences of all OFs decreased with increasing age. The initial increase in OF prevalence with increasing age can be attributed to higher prevalence of preexisting comorbidities and organ dysfunctions with higher age [5]. Furthermore, we detected parallel increasing trends of ISS with OF prevalences until the age cohort 80–84, followed by decreasing injury severity among oldest age groups as a consequence of lower prevalence of high energy trauma in subgroups older than 84 years [27]. A multivariate analysis performed by Cole et al. showed that frailty and ISS, but not age correlate with risk of MOF and therefore supports our finding of missing correlation of age and OF prevalence [8].

Although males have comparable prevalences of comorbidities [28], OF and MOF was more common among male than female EMTP. This is in line with several other studies, which showed that the male sex is an independent risk factor for developing OF and MOF [25, 28–31]. Several studies showed that the acute phase parameter Interleukin-6 (IL-6) after trauma is significantly higher in males than in females [31–33] resulting in hyperinflammation including systemic inflammatory response syndrome (SIRS) and sepsis, finally ending up in organ dysfunction, OF or MOF [31, 32, 34, 35].

OF can result from a direct injury of the affected organ, like contusion, bleeding or rupture, or can be a consequence of indirect damage like hypoperfusion, ischaemia or hyperinflammation. As such, patients suffering from CNS failure sustained MAIS ≥ 3 head injuries in 83.9% of all CNS failure cases making it the most common isolated OF in this study. Unlike CNS failure, other OFs were less frequently associated with severe injuries of the same body region. Only 45.4% of all cardiovascular failure patients and 50.6% of all lung failure patients sustained MAIS ≥ 3 thorax injuries and serious extremity trauma (MAIS ≥ 3) occurred more frequently than serious abdominal trauma in liver or renal failure patients. As serious direct injuries of heart, liver, both lungs or both kidneys are a rare entity among mostly low-impact blunt trauma of EMTP, this shows that most failures of these organs are caused by indirect damage like hypoperfusion and haemorrhage [9, 36–38], dysregulation of the inflammatory system, like SIRS and sepsis [9, 31, 32, 34–36, 39], or other pathophysiological mechanisms, like coagulopathy [35, 39], rhabdomyolysis [36, 37, 39] or acid-base-disbalances [9, 38, 40]. Especially cardiovascular failure is the most frequently detected OF in MOF, because reduced organ perfusion can impair other organs and can trigger a chain reaction resulting in failure of multiple organs [9, 35–38].

Because mortality rates of different OFs differ significantly, we analysed mortality rates of distinct combinations of two OFs in MOF and detected highest mortality for the combination of CNS and liver failure (87.5%), as these organs also showed highest mortality in single OF, too. On the one hand, a systematic review conducted by van Breugel et al. reported traumatic brain injury (TBI) as the most common reason for death after trauma [41], and on the other hand, liver failure can influence several other organ systems and is associated with high complication rates resulting in disproportionately high mortality rates [38, 42]. Because most CNS failures are a consequence of intracranial bleeding, haemostatic failure in severe head injuries is associated with progression of intracranial haemorrhagic lesions and therefore aggravates the severeness of CNS failure resulting in the second deadliest OF combination (68.5%) [43].

As 37.0% of all EMTP sustain MOF or OF and 80.9% of all non-survivors suffered from MOF (51.1%) or OF (29.8%), this highlights the relevance of screening for and specific treatment of OF and MOF. Especially in EMTP with more severe injuries a close monitoring of OF and MOF is important, as we detected a significant correlation of ISS and number of affected organs (*r* = 0.44, *p* < 0.001). This is in line with several other studies, which also detected a correlation of ISS and risk of MOF development [8–10, 25]. With increasing numbers of organ failures mortality rises significantly with mortality rates of 7.4% in non-OF, 45.0% in OF and 59.0% in MOF patients. These mortality rates increased continuously with increasing age with a steeper increase of mortality in OF and MOF patients compared to non-OF patients highlighting that MOF and OF impact on mortality is higher in older EMTP. Therefore, we detected a mortality rate of 94.8% in the 94–99 years old MOF subgroup. Moreover, differences in mortality between MOF and OF patients become smaller with increasing age in this study, what also underlines that older patients have less physiological reserves to sustain OF and MOF. In line with this the detected decreasing LoS at ICU both in survivors and non-survivors with increasing age further supports that older patients have less physiological reserves, which was also reported by Cole et al. [8]. Considering only data of patients, who passed away, we detected that mean ISS in older non-survivors was lower as another indicator for less capacities to survive longer at ICU. Therefore, especially older EMTP and EMTP with a higher ISS should be monitored and treated carefully to prevent OF, MOF and death.

Furthermore, the status of recovery after survival of OF and MOF was also clearly worse than the recovery status of EMTP without OF or MOF, as MOF patients had an approximately 21-times higher and OF patients 11-times higher risk of discharge in a permanent comatose state compared to non-OF patients (GOS 2: non-OF: 0.5%, OF: 5.3%, MOF: 10.6%). While the majority of non-OF EMTP were discharged without the need for daily care (GOS ≥ 4: 92.8%), 60.4% of OF/MOF survivors (GOS ≥ 4: MOF: 50.1%; OF: 70.5%) achieved a good recovery state. As shown by our study and other publications, on the one hand modern major trauma care can rehabilitate the vast majority of EMTP to a good functional status upon discharge [8, 13, 44]. On the other hand, OF and MOF not only rise mortality significantly but also have a significant impact on the recovery status of EMTP.

### Impact of (multiple) organ failure on mortality

Comparing the different OFs with each other showed that liver failure was the most relevant risk factor for mortality in this study (OR: 5.13, *p* < 0.001), most likely because patients suffering from liver failure showed higher ISS and suffer more often from complications like sepsis [38, 42]. Due to the complex effects of liver failure on other organ systems, liver failure occurs in most cases as a part of MOF (97.2% of liver failure patients showed MOF in this study) [8, 9, 25]. This resulted in highest OR and mortality rate among all OFs, as well as second longest mean LoS at ICU (11 days).

Another OF with high mortality and limited therapeutic options is CNS failure and therefore, calculated OR for CNS failure is 3.63 in this study. Although brain injuries are in general the most frequently documented cause of death after major trauma [41], OR of CNS failure is lower than OR of liver failure, because the RISC II score considers severe head injuries (MAIS ≥ 3) additionally and therefore takes an important risk factor for CNS failure into account [17]. Nevertheless, OR of CNS failure remains high, because CNS failure is not always a direct consequence of severe head injury [41].

Although there are good therapeutic options for renal failure and due to renal replacement therapy long-term survival with persistent renal failure is possible, OR (3.25), overall mortality (59.8%) and LoS at ICU (10 days) of renal failure are the third highest. This is in line with a systematic review conducted by Søvik et al., as they reported an mortality of severe acute kidney injury (AKI) of 45% according to RIFLE (Risk, Injury, Failure, Loss of kidney function, and End-stage kidney disease) / AKIN (acute kidney injury network-criteria) / KDIGO (Kidney Disease: Improving Global Outcomes), which definitions are quite comparable to a SOFA score 3 and 4 [36]. As several authors mentioned age as a relevant risk factor and Søvik et al. considered all adults in their study, a higher mortality rate in our study is consistent [36, 39, 45].

Cardiovascular failure showed the highest prevalence of all OFs in this study and an OR of 1.95. It is usually caused by haemorrhagic shock and preexisting cardiovascular diseases increase mortality risk significantly [46–48]. Furthermore, secondary cardiovascular failure after haemorrhagic shock also increases late mortality, because a reduced cardiac output in the days after trauma can cause several other OFs with consecutive highest prevalence of all OFs in MOF patients [39, 48, 49].

Lung failure was both the third frequent OF in MOF and as singular OF. Besides liver failure, lung failure showed highest rates of co-incidence with other OFs, as an acute respiratory distress syndrome (ARDS) frequently occur in MOF patients [44]. According to van Wessem et al. only 7% of major trauma patients suffering from ARDS die of ARDS [44]. As ARDS patients often need a prolonged ventilation [44], this resulted in the longest mean ICU LoS of lung failure patients within this study (12 days). Therefore, lung failure is on the one hand the most impactful OF for a prolonged stay at ICU but have on the other hand only a minor impact on mortality (OR: 1.16, *p* = 0.010).

Haemostatic failure was neither associated with a positive or negative impact on mortality (OR: 1.07; *p* = 0.299). The detected mortality rate of haemostatic failure is way lower in isolated OF compared to mortality rate in MOF, because haemostatic failure is often a consequence of haemorrhagic shock and therefore combination with cardiovascular failure as an effect of acute haemorrhage is common, resulting in higher overall mortality [50, 51]. It is important to mention, that SOFA score defines haemostatic failure solely by platelets counts and therefore does not reflect the whole complexity of haemostasis, which may result in potential imprecisely diagnosing of haemostatic failure [52].

MOF and sepsis showed negative ORs in the multivariate logistic regression (MOF: OR: 0.82, *p* = 0.013; sepsis: OR: 0.85, *p* = 0.016). Of course, both sepsis and MOF are important risk factors for mortality after major trauma and indeed we detected a high mortality among MOF patients in this study [8–10, 53], but an OR < 1 for MOF in this multivariate regression shows that MOF dependent mortality risk is lower than the simple summation of risks of the involved OFs. As OR of sepsis was also < 1 in this study, this indicates that septic OF seems to be less lethal than trauma induced OF.

### Limitations

As the TR-DGU provide only limited data about the patients’ pre-injury status, we were unable to assess the patients’ frailty status, which predicts mortality risk significantly better than age or ASA. Therefore, we could not evaluate whether specific comorbidities are associated with a higher risk of specific OFs. Furthermore, treatment algorithms between the different trauma centres likely varied. To minimize such differences based on different trauma systems, we only included trauma centres associated with the German TraumaNetzwerk DGU^®^.

Deaths within the first 24 h are normally a consequence of pre-existing factors and trauma severity, whereas deaths within the subsequent phase are mostly caused by organ dysfunction/failure, sepsis, and other complications [35, 53]. Because the TR-DGU did not differentiate between the first and the following days, we differentiated between resuscitation phase and ICU phase and consequently included solely patients, who survived the initial resuscitation and were admitted to ICU.

As the TR-DGU does not provide information about the length and the exact timepoint of a distinct OF it remains unclear whether the different OFs existed at the same timepoint or after each other. Nevertheless, several other studies also defined MOF as the failure of at least two organs without a time relation [9, 13, 53–55]. Although the SOFA score is one of the best validated scores for organ dysfunction and failure, it has its limitations in OF and MOF assessment, as breaking down the complex pathobiology of OFs to one criterion is limited in precision [56]. Nevertheless, Fröhlich et al. showed that the SOFA score is the most balanced score for diagnosing MOF in major trauma patients in terms of sensitivity and specificity in comparison to the Marshall Multiple Organ Dysfunction Score (MODS) and Denver Post Injury Multiple Organ Failure Score [54].

The primary endpoint of this study was inpatient mortality or 30-days-mortality in patients with a hospital LoS of > 30 days, while a secondary endpoint was the recovery status upon discharge from hospital. Although both endpoints miss important long-term effects of trauma and OF/MOF, date of discharge or thirty days after trauma are reasonable timepoints, as general 1-year mortality rise with increasing age and therefore non-trauma- or non-OF/MOF-related deaths would distort the results [57]. Unfortunately, we could not analyse the cause of death, as the TR-DGU do not provide this data, although cause of death analysis would have provided further insights into whether and how OF and MOF contributed to patients’ death. Especially in elderly patients, decisions to withdraw or limit interventions are common, but the impact of such decisions on the incidence of OF/MOF and effects on mortality and morbidity are difficult to assess and are not further specified in the TR-DGU. As there is no data about geriatric-co-management, long-term follow-up, type of care after discharge, and further rehabilitation provided in the TR-DGU further studies are crucial to evaluate impacts of specific treatment strategies and their long-term effects.

## Conclusion

This study evaluated prevalences, recovery status and mortality risks of distinct OFs and MOF in EMTP and identified contributing factors to OF and MOF development. OF and MOF are a huge challenge in the treatment of EMTP, as prevalence of OF and MOF patients are high among elderly patients (OF: 16.0%; MOF: 21.0%) and OF and MOF are associated with high mortality rates (non-OF: 7.4%, OF: 45.0%, MOF: 59.0%) and impaired recovery status among survivors (GOS 2 or 3: non-OF: 7.2%, OF: 29.6%, MOF: 50.0%). Although incidences of specific OFs showed a bell-shaped curve with decreasing incidence among the oldest patients, mortality of OF and MOF rises with increasing age with mortality rates up to 72.4% in OF patients aged 90–94 years and 94.8% in MOF patients aged 95–99 years. 80.9% of all EMTP died with failure of at least one organ and 51.1% died with MOF. Due to limited success in treatment options of CNS and liver failure, these OF as well as renal failure were the most life-threatening OFs in this study.

Based on these findings clinicians should be aware that OF and MOF occur frequently in EMTP and that these patients have a substantial risk of dying. Especially older patients, patients with a higher ISS and patients suffering from liver failure in combination with CNS or haemostatic failure are at highest mortality risk. Therefore, admission of EMTP to ICU should be performed liberally and a close screening for OF and MOF is essential to detect signs of OF as early as possible. Early and aggressive treatment of OF and MOF in EMTP appears to be the key strategy to lower mortality of EMTP, who survived the initial resuscitation phase.

## Supplementary Information

Below is the link to the electronic supplementary material.

Supplementary Material 1

## Data Availability

The data that support the findings of this study are available from the corresponding author upon reasonable request.
