# Supplementary material for: Older age and post-traumatic organ failure a TraumaRegister DGU® analysis of 34,469 elderly major trauma patients
Source: Eur J Trauma Emerg Surg. 2026 Jun 15;52(1):189. doi: 10.1007/s00068-026-03240-2 (PMC13269517; doi:10.1007/s00068-026-03240-2)
Supplement: Supplementary file 1 — Supplementary Material 1 [file 68_2026_3240_MOESM1_ESM.docx]

# Supplemental File

# Older Age and Post-Traumatic Organ Failure

# –

# A TraumaRegister DGU® Analysis of 34,469 Elderly Major Trauma Patients

Tim Niklas Bewersdorf ^1,2,*^, Rolf Lefering ^3^, Stephan Stein ^2^, Jan Streblow^2^, Sebastian Findeisen ^2^, Gerhard Schmidmaier ^2^ and Tobias Grossner ^2^

^1^ Faculty of Medicine, Heidelberg University, 69120 Heidelberg, Germany

^2^ Heidelberg Trauma Research Group, Clinic for Trauma and Reconstructive Surgery, Centre for Orthopaedics, Trauma Surgery and Spinal Cord Injury, University Hospital Heidelberg, 69120 Heidelberg, Germany.
^3^ Institute for Research in Operative Medicine (IFOM), University of Witten / Herdecke, Cologne, Germany.

## TraumaRegister DGU®

The general objective of the multi-centre registry TraumaRegister DGU® (TR-DGU) is, since 1993, to gather pseudonymised and standardised pre-clinical and clinical data of severely injured trauma patients. The data collection process is organized prospectively and spans across four distinct phases, beginning with the preclinical phase at the scene of the accident, followed by the emergency resuscitation phase in the trauma resuscitation room (TRR) including the initial surgical care. The third phase includes treatment at the intensive care unit (ICU), while the fourth and final phase collects data regarding the discharge of the patient. For each patient, detailed information is recorded, such as demographic characteristics, injury patterns, pre-existing comorbidities, as well as all preclinical treatment and treatment in the TRR, operating theatre and at ICU. The documentation also covers important laboratory results, blood transfusions, complications and the outcome of each individual. All trauma patients admitted to the hospital via TRR with subsequent ICU care or trauma patients reaching the hospital with vital signs but die before admission to ICU are documented within the TR-DGU. Patients can be reported in the register by using the basic dataset, or the more detailed standard datasets. The standard dataset provides further information about preclinical findings, extended diagnostics within the resuscitation phase, diagnostics and therapies at ICU, as well as information about OF/MOF and other complications and outcome parameters.

The infrastructure for data entry, management, and analysis is provided by the AUC – Academy for Trauma Surgery (AUC – Akademie der Unfallchirurgie GmbH), which is affiliated with the German Trauma Society. Scientific leadership is ensured by the Committee on Emergency Medicine, Intensive Care and Trauma Management (Sektion NIS) of the German Trauma Society. Participating hospitals submit pseudonymized patient data of all trauma patients, who met the inclusion criteria, to a central database using a web-based platform. In general, the data quality of the entered data is high, and rate of missing data is low [19]. All scientific analyses of the data must be approved through a peer review process, as specified in the publication guidelines of the TR-DGU.

While most participating hospitals are located in Germany (about 90%), an increasing number of institutions from other countries are also contributing data. Currently, the database receives information on more than 30,000 cases per year from almost 700 hospitals, resulting in 397,910 patients between 2014 and 2023, which was the study period of this analysis. Participation in the TR-DGU is generally voluntary, but for hospitals that are part of the TraumaNetzwerk DGU®, submitting at least a basic dataset is mandatory for quality assurance purposes.
